# Optimization of a Human Bacille Calmette-Guérin Challenge Model: A Tool to Evaluate Antimycobacterial Immunity

**DOI:** 10.1093/infdis/jiv482

**Published:** 2015-10-08

**Authors:** Alice Minhinnick, Stephanie Harris, Morven Wilkie, Jonathan Peter, Lisa Stockdale, Zita-Rose Manjaly-Thomas, Samantha Vermaak, Iman Satti, Paul Moss, Helen McShane

**Affiliations:** 1 The Jenner Institute, University of Oxford; 2 School of Cancer Sciences, University of Birmingham, United Kingdom

**Keywords:** BCG, human mycobacterial challenge model, vaccine effectiveness, tuberculosis, anti-mycobacterial immunity

## Abstract

***Background.*** There is an urgent need for an improved tuberculosis vaccine. The lack of a validated correlate of protection slows progress in achieving this goal. A human mycobacterial challenge model, using bacille Calmette-Guérin (BCG) as a surrogate for a *Mycobacterium tuberculosis* challenge, would facilitate vaccine selection for field efficacy testing. Optimization of this model is required.

***Methods.*** Healthy BCG-naive adults were assigned to receive intradermal standard-dose BCG SSI (group A), standard-dose BCG TICE (group B), high-dose BCG SSI (group C), and high-dose BCG TICE (group D). Two weeks after BCG challenge, skin biopsy of the challenge site was performed. BCG mycobacterial load was quantified by solid culture and quantitative polymerase chain reaction.

***Results.*** BCG was well tolerated, and reactogenicity was similar between groups, regardless of strain and dose. There was significantly greater recovery of BCG from the high-dose challenge groups, compared with standard-dose challenge. BCG strain did not significantly affect BCG recovery.

***Conclusions.*** BCG challenge dose affects sensitivity of this model. We have selected high-dose BCG SSI to take forward in future challenge studies. Assessment of candidate tuberculosis vaccine effectiveness with this optimized model could contribute to vaccine selection for efficacy trials.

***Clinical Trials Registration.*** NCT02088892.

The quest for an effective tuberculosis vaccine is a public health emergency [1]. There is a critical need for a vaccine that can provide greater and more-consistent protection against tuberculosis than that offered by the only licensed vaccine, bacille Calmette-Guérin (BCG). This is especially important in an era that sees an estimated 9.0 million incident tuberculosis cases and 1.5 million tuberculosis-related deaths each year, exacerbated by an increasing burden of antimicrobial resistance [1].

The lack of reliable and validated immunological correlates of protection hampers the development of tuberculosis vaccines. In the absence of measureable markers to predict candidate vaccine effectiveness, the field has so far relied on animal challenge models and in vitro functional assays that assess the ability of a vaccine to inhibit mycobacterial growth (such as the mycobacterial growth indicator tube [MGIT] [2]). It is unclear how reliably these models forecast in vivo human efficacy. Selecting the best or most-appropriate candidate tuberculosis vaccines for further investment, research, and development is difficult. A safe and relevant mycobacterial human challenge model to allow more-rapid early assessment of candidate tuberculosis vaccines could be a game changer.

In general, large and expensive phase 2b and 3 field efficacy trials are required to demonstrate whether safety and immunogenicity results from small phase 1 trials translate into an impact on overall disease burden. Developers of vaccines, including those targeting pathogens responsible for malaria and typhoid [3, 4], have adopted human challenge models to more quickly and inexpensively assess vaccine effectiveness, helping to rationalize which vaccines progress to field efficacy trials. Successful use of human challenge studies has accelerated advancement in these vaccine development pipelines. Safety and ethical reasons preclude the use of *Mycobacterium tuberculosis* in a human mycobacterial challenge model.

We have evaluated an alternative approach: the *Mycobacterium bovis* BCG challenge model. The model is based on the hypothesis that a tuberculosis vaccine that successfully reduces replication of *M. tuberculosis* should also reduce BCG replication. BCG is a potentially useful surrogate of *M. tuberculosis* for a challenge model. Years of use as a licensed vaccine verify its good safety record [5, 6]. BCG, when administered intradermally, causes a self-contained and limited infection. Importantly, the CD4^+^ T-cell mediated immune responses elicited by both mycobacteria are very similar [7]. Although there are some *M. tuberculosis* antigens not present in BCG, which, if selected as tuberculosis vaccine candidates, would not be expected to have an impact on BCG replication, the optimization of a BCG human challenge model can still establish the clinical parameters for subsequent challenge models using attenuated *M. tuberculosis* strains. We previously demonstrated that BCG-vaccinated mice that were later challenged with intradermal BCG had suppressed mycobacterial growth that mimicked observations following intranasal *M. tuberculosis* challenge, suggesting that a skin mycobacterial challenge may adequately reflect a vaccine effect in the lung [8].

We have recently applied this BCG challenge model to humans. Our pilot study of a human BCG challenge model demonstrated that the degree of growth suppression of BCG can be measured in a punch biopsy specimen the skin from the challenge site where BCG was intradermally administered. We showed that the live mycobacterial load can be quantified up to 1 month after challenge [9].

We found that the model can distinguish between BCG-naive and BCG-vaccinated groups [10], suggesting that prior BCG vaccination gives some protection against a subsequent challenge dose, in a population in which BCG has been shown to be protective [11]. We have also assessed this BCG challenge model in individuals administered the candidate vaccine MVA85A [12]. We found that MVA85A receipt prior to BCG challenge had no effect on the subsequent recovery of BCG, a finding that may be consistent with the results of the recent infant MVA85A efficacy trial or, alternatively, a reflection of the limitations in the model's sensitivity to date [10, 13]. However, a major limitation of the model to date has been low mycobacterial readouts that approached the lower limit of detection, reducing both the sensitivity and ability to detect inter-individual variation in BCG suppression [10].

In this study, we evaluated the effect of both BCG strain and dose on subsequent mycobacterial recovery, with a view to improving model sensitivity and the ability to discriminate between individuals with differing levels of vaccine-induced antimycobacterial immunity. We compare the use of 2 licensed strains of BCG at 2 different doses, to select the most-suitable conditions for BCG challenge for the future testing of tuberculosis vaccine efficacy.

## METHODS

### Study Design and Participants

We undertook a controlled human challenge study using 2 different licensed strains of BCG at standard dose or high dose (defined as 3 times the standard dose). This study was conducted in accordance with the Declaration of Helsinki and good clinical practice. The National Research Ethics Service South Central–Oxford B research ethics committee reviewed and approved the study (REC reference 14/SC/0036). We conducted this study at 2 trial sites, recruiting volunteers at the Centre for Clinical Vaccinology and Tropical Medicine, Oxford, and the National Institute for Health Research Wellcome Trust Clinical Research Facility, Birmingham (clinical trials registration NCT02088892).

**Figure 1.**
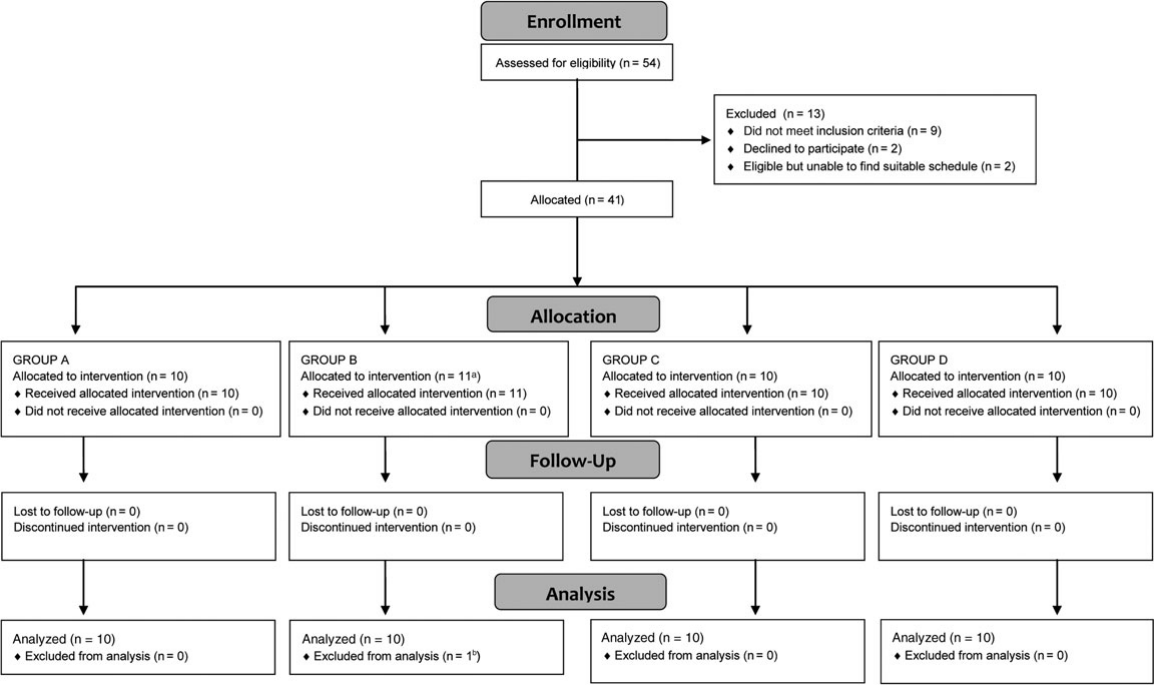
Study profile. CONSORT (Consolidated Standard of Reporting Trials) flow diagram, showing volunteer recruitment and follow up. Volunteers were allocated to groups A and B in parallel. Once enrollment was completed for both groups, subjects were enrolled in groups C and D. ^a^A replacement volunteer was required in group B because it was unclear whether bacille Calmette-Guérin (BCG) had been administered intradermally or subcutaneously in a volunteer who had no signs of local inflammatory response to the BCG challenge, resulting in 11 volunteers who received the group B intervention; ^b^The group B volunteer who lacked a local response to BCG was excluded from analysis, resulting in 10 volunteers for analysis in group B. This volunteer was followed up for 28 days after challenge, and there were no safety concerns.

All 41 volunteers gave written informed consent before participation. We initially recruited 40 BCG-naive volunteers (groups A–D). One volunteer was excluded following challenge and replaced, yielding 40 volunteers for analysis (Figure 1).

All volunteers were healthy, aged 18–55 years, and BCG naive. The full inclusion and exclusion criteria are described in Supplementary Methods 1. All enrolled volunteers had normal baseline hematology and biochemistry findings and were negative for hepatitis B and C viruses and human immunodeficiency virus. Latent *M. tuberculosis* infection was excluded on the basis of negative findings of a negative T-spot.*TB* test (Oxford Immunotec).

A multiarm study design was used to allow simultaneous comparison of 2 conditions: BCG strain and BCG dose. We allocated volunteers into 4 groups: standard-dose BCG SSI (group A), standard-dose BCG TICE (group B), high-dose BCG SSI (group C), and high-dose BCG TICE (group D). Because there was no perceived benefit to blinding, for logistical reasons volunteers were allocated to groups, with as many volunteers as possible allocated to the same group on a given day. Once groups A and B were fully enrolled, volunteers were allocated to groups C and D. In each of groups C and D, a 2-week window elapsed between the challenge of the first and second volunteers and between the second and subsequent volunteers, to permit the detection of any unexpected adverse events associated with high-dose BCG.

### Challenge and Follow-up

Volunteers were challenged intradermally with an injection of BCG (0.15 mL) into the upper arm. The desired dose range of BCG was achieved by serial dilution as described in Supplementary Methods 2. Group A volunteers were challenged with 2–8 × 10^5^ colony-forming units (CFU) of BCG SSI, group B volunteers received 2–8 × 10^5^ CFU of BCG TICE, group C volunteers received 6 × 10^5^–2.4 × 10^6^ CFU of BCG SSI, and group D volunteers received 6 × 10^5^–2.4 × 10^6^ CFU of BCG TICE.

To reduce the variation in challenge dose within each group, we challenged as many volunteers as possible with BCG from the same vaccine vial and within 2 hours of reconstitution. The challenge dose was confirmed by plating serial dilutions of a 100-µL aliquot of the BCG strain onto solid Middlebrook 7H11 agar (Appleton Woods).

After challenge, we followed up all volunteers for 1 month, with clinic visits on days 2, 7, 14, and 28 after BCG challenge. On day 14, the volunteers underwent punch biopsy of the challenge site. Blood samples were collected at days 0, 2, 7, and 14, and peripheral blood mononuclear cells (PBMCs) were isolated and cryopreserved.

We obtained data on the incidence of solicited and unsolicited local (challenge site) and systemic adverse events, reported by volunteers on diary cards for 14 days after BCG challenge, and by direct questioning at clinic follow-up appointments on days 2, 7, and 14. Challenge sites were assessed for local reactions, and vital signs were recorded. The day 28 follow-up was for clinical review of the BCG challenge and biopsy site. Adverse events were assessed by the trial investigators to determine their relationship to BCG challenge. These adverse events were classified as unrelated, possibly related, probably related, or definitely related to BCG challenge.

### Skin Biopsies

We performed the punch biopsy using a sterile technique with a standard 4-mm punch (Stiefel); 0.5–3.4 mL of 1% lignocaine was infiltrated subcutaneously. The punch biopsy specimen was collected from the center of the BCG vaccination site, snap frozen on dry ice, and stored in liquid nitrogen until processing.

### Homogenization and Culture of Biopsy Specimens

All 40 biopsy specimens from volunteers in groups A–D were processed on the same day. Each biopsy specimen was thawed in a 37°C water bath and transferred to a Dispomix tube (MACS) containing 1 mL sterile phosphate buffered saline (PBS). Tubes were loaded onto a Dispomix machine (Thistle Scientific) and homogenized as previously described [8]. A total of 100 µL of neat homogenate and 100 µL of a 10^−1^ dilution, in sterile PBS, were plated in triplicate onto Middlebrook 7H11 agar. For groups C and D (who received a high-dose challenge), a 10^−2^ dilution was also performed. BCG SSI and BCG TICE vaccine vials were reconstituted in PBS, and 100 µL of appropriate dilutions were plated in triplicate as positive controls. Plates were incubated at 37°C for 4 weeks before counting. The remaining biopsy specimen homogenate was stored at −80°C for later DNA extraction.

### DNA Extraction

Biopsy specimen homogenate was thawed, and BCG DNA from 200 µL of homogenate was released using the tough microorganism lysing kit (Precellys) in a Precellys24 machine at 6500 rpm for 3 × 30 seconds. Homogenate was transferred to a separate tube and 50 µL PBS used to wash remaining homogenate from the beads. A total of 180 µL of ATL buffer and 20 µL of proteinase K (Qiagen) were added, and the homogenate was vortexed and incubated at 56°C for 4 hours. Following this step, extractions were performed as previously described [8].

### Quantitative Polymerase Chain Reaction

Primers ET 1/3 (forward: CCG CCG ACC GAC CTG ACG AC; reverse: GGC GAT CTG GCG GTT TGG GG), modified by Minassian et al [8], were used for detection of BCG DNA. These are complementary to regions flanking the BCG deletion sequence, RD1, and amplify a 196–base pair fragment [14]. Polymerase chain reaction (PCR) analyses were performed as previously described [8]. A standard curve was obtained by extracting BCG DNA from 1:10 serial dilutions of 5 pooled SSI vaccine vials in PBS and correcting for live BCG from the corresponding CFU counts on solid agar.

### Ex Vivo Interferon γ (IFN-γ) Enzyme-Linked Immunospot (ELISpot) Assay

ELISpot assays were performed, as previously described [15], on freshly isolated PBMCs from all volunteers on the day of challenge (day 0) and the day of biopsy (day 14). Responses to purified protein derivative (PPD) from *M. tuberculosis* (strain SSI; 20 µg/mL) were assessed for all volunteers at both time points. *Staphylococcus* enterotoxin B (Sigma) was used as a positive control (10 µg/mL). Unstimulated PBMCs were used as a measure of background IFN-γ production. Results are reported as spot-forming cells per million PBMCs, calculated by subtracting the mean count of the unstimulated PBMCs from the mean count of triplicate antigen wells and correcting for number of PBMCs in the well.

### Statistical Analysis

Statistical analyses were performed using GraphPad Prism. One-way analysis of variance (Kruskal–Wallis) and Mann–Whitney *U* tests were used to determine significant differences between groups. The Wilcoxon matched-pairs test was used to determine differences between time points in the same group. The Spearman rho test was used to determine correlations between numbers of BCG recovered from biopsy specimens and ex vivo IFN-γ ELISpot responses.

## RESULTS

Between 10 March 2014 and 11 September 2014, 41 of 54 volunteers screened for eligibility were challenged with BCG (Figure 1). One volunteer who did not develop any local reaction to BCG was excluded after challenge because it was likely that the administration of BCG had been subcutaneous rather than intradermal. This volunteer was followed up for 14 days, and there were no safety concerns. They were excluded from analysis and replaced with a new volunteer.

Demographic and baseline clinical characteristics of the study participants were similar between groups (Table 1). A list of the frequency and maximum severity of adverse events assessed as possibly, probably or definitely related to challenge is provided in Table 2.

**Table 1.** Baseline Demographic Characteristics of Study Participants, by Group

Characteristic	Group A (n = 10)	Group B (n = 10)	Group C (n = 10)	Group D (n = 10)
Age, y, mean (range)^a^	25.7 (18–35)	28.1 (20–39)	23.1 (19–28)	30.0 (18–44)
Female sex	6	5	8	8
Place of birth				
Europe	9	10	9	8
Australasia	1	0	1	1
Africa	0	0	0	1

Data are no. of participants, unless otherwise indicated.

^a^ Values were compared by 1-way analysis of variance, with the Tukey test for multiple comparisons. No significant difference was observed between groups (*P* > .05).

**Table 2.** Maximum Local and Systemic Adverse Events ≤14 Days After Bacille Calmette-Guérin Challenge, by Group

Symptom, Intensity	Group A (n = 10)	Group B (n = 10)	Group C (n = 10)	Group D (n = 10)	Overall (n = 40)
Local					
Pain					
Mild	6	7	7	5	25 (62.5)
Moderate	0	0	1	0	1 (2.5)
Severe	0	0	0	0	0
Redness					
Mild	10	9	8	9	36 (90)
Moderate	0	1	2	1	4 (10)
Severe	0	0	0	0	0
Swelling					
Mild	10	10	10	10	40 (100)
Moderate	0	0	0	0	0
Severe	0	0	0	0	0
Warmth					
Mild	8	6	4	9	27 (67.5)
Moderate	0	0	1	0	1 (2.5)
Severe	0	0	0	0	0
Itch					
Mild	6	7	6	7	26 (65)
Moderate	1	0	0	1	2 (5)
Severe	0	0	0	1	1 (2.5)
Scaling	7	6	5	6	24 (60)
Lymphadenopathy	0	0	0	0	0
Systemic					
Feverishness					
Mild	2	0	1	2	5 (12.5)
Moderate	0	0	1	0	1 (2.5)
Severe	0	0	0	0	0
Temperature					
Mild	0	0	0	0	0
Moderate	0	0	1	0	1 (2.5)
Severe	0	0	0	0	0
Headache					
Mild	4	1	3	4	12 (30)
Moderate	1	1	1	2	5 (12.5)
Severe	1	0	1	0	2 (5)

Data are no. or no. (%) of volunteers, counted once at the time of the highest severity grading of the event. All events were confirmed by trial investigators as possibly, probably, or definitely related to BCG challenge.

**Figure 2.**
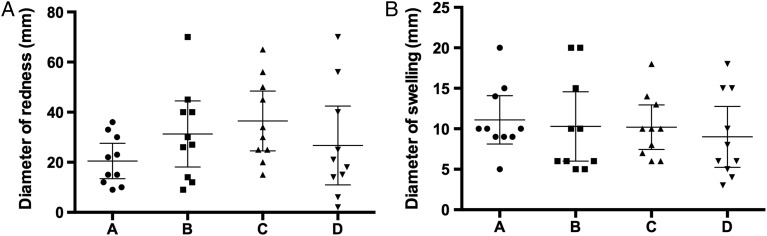
Local reactogenicity. Maximum diameters of redness (*A*) and swelling (*B*) at the challenge site, with mean values and 95% confidence intervals. Mean values were compared with 1-way analysis of variance, with the Tukey test for multiple comparisons. The difference in mean diameter was not statistically significant between groups (*P* > .05). Dots represent values for individual volunteers.

BCG challenge was well tolerated. All participants developed an expected local inflammatory reaction, regardless of which dose and strain they were administered. Local adverse events were comparable between groups and were generally mild. There was no difference between the diameter of redness or swelling across the different groups (Figure 2). When visually assessing the photographed challenge sites, a blinded clinician was unable to correlate severity of local reaction to challenge group. The majority of reported systemic adverse events were mild, and the mean number of systemic adverse events per person did not differ significantly between groups. The proportion of volunteers who experienced at least 1 systemic adverse event related to vaccination was broadly similar between groups: 6 of 10 volunteers in group A, 2 of 10 in group B, 5 of 10 in group C, and 7 of 10 in group D (Table 2). Although 6 volunteers (2 in each of groups A, C, and D) reported feelings of feverishness, only 1 of these volunteers, who was in group C, had a documented increase in temperature that was deemed related to challenge (38.6°C on day 2 and 37.8°C on day 10). No serious adverse events occurred.

### BCG Challenge Dose

Groups A and B received standard dose (2–8 × 10^5^ CFU) BCG SSI and BCG TICE, respectively. Four vaccine vials were used in each group to challenge the 10 volunteers. The range in dose in group A was 1.84 × 10^5^–3.31 × 10^5^ CFU (median, 2.28 × 10^5^ CFU) and in group B was 1.09 × 10^5^–2.84 × 10^5^ CFU (median, 2.35 × 10^5^ CFU). Groups C and D received 3 times the standard dose of BCG SSI and BCG TICE, respectively. Six vaccine vials were used in group C and 7 vials were used in group D to challenge the 10 volunteers within the group. The range in dose received was much wider for these high-dose groups, with 7.4 × 10^5^–1.38 × 10^6^ CFU (median 9.86 × 10^5^ CFU) used in group C and 4.45 × 10^5^–4.32 × 10^6^ CFU (median, 7.5 × 10^5^ CFU) used in group D.

### High-Dose Challenge Results in Significantly Greater Numbers of BCG Recovered Than Standard-Dose Challenge

**Figure 3.**
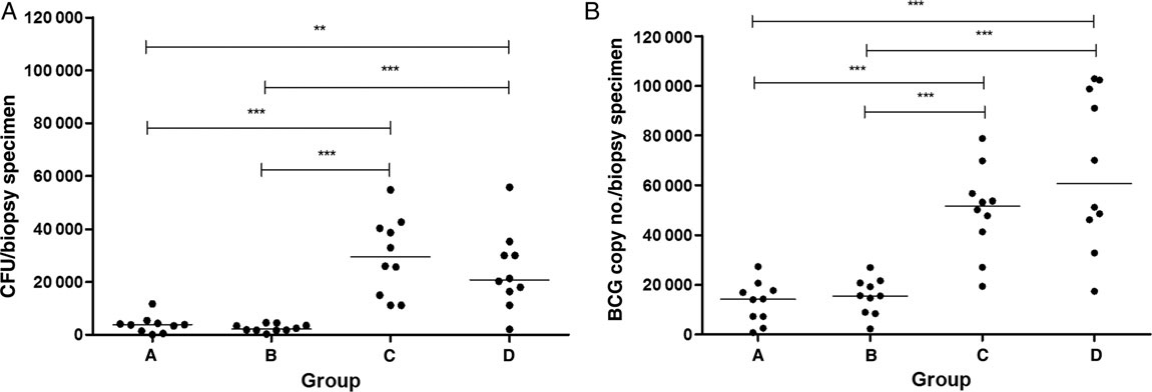
Recovery of bacille Calmette-Guérin (BCG) by culture on solid agar (*A*), and BCG quantitation by quantitative polymerase chain reaction (*B*) in skin biopsy specimens obtained from 40 healthy volunteers challenged with standard-dose BCG SSI (group A) or BCG TICE (group B) or high-dose BCG SSI (group C) or BCG TICE (group D). Dots represent individual volunteers, and black lines show median values. **P < .01 and ***P < .00, by the Mann–Whitney *U* test. Abbreviation: CFU, colony-forming units.

BCG was detected in all 40 biopsy specimens, by both culture on solid agar and quantitative PCR (qPCR), and a significant positive correlation was observed between the 2 methods of quantification (*r* = 0.6643 and *P* < .0001, by the Spearman rho test; data not shown). qPCR values were on average higher than CFU counts, owing to qPCR detecting dead as well as live bacteria. There was no difference in the number of BCG recovered between the groups who received the same challenge dose; that is, there was no difference between groups A and B or C and D and therefore no difference in number recovered between strains of BCG. However, the number of BCG detected in the high-dose groups (C and D) was significantly greater than that detected in the low-dose groups, by both methods of quantification (*P* < .05, by the Mann–Whitney *U* test; Figure 3). Correcting CFU counts for challenge dose received did not change the statistical significance of the differences between the groups (data not shown). When the number of CFU detected was corrected for biopsy specimen weight and expressed per gram of tissue (data not shown), group B had significantly greater CFU counts than group A (*P* = .0288, by the Mann–Whitney *U* test), but the differences between the other groups remained unchanged. When all groups were combined, there was a significant positive correlation between challenge dose and CFU count in biopsy specimens (*r* = 0.7494 and *P* < .0001, by the Spearman rho test; Supplementary Figure 1). This correlation was lost when the groups were broken down into standard-dose and high-dose challenge groups (*r* = 0.0502 and *P* = .8335 for the standard-dose groups and *r* = 0.3207 and *P* = .1807 for the high-dose groups, by the Spearman rho test). Volunteers who were challenged from the same vaccine vial, and who therefore received the same challenge dose, had varying CFU counts in biopsy specimens (Supplementary Figure 1).

### Ex Vivo IFN-γ ELISpot Responses

**Figure 4.**
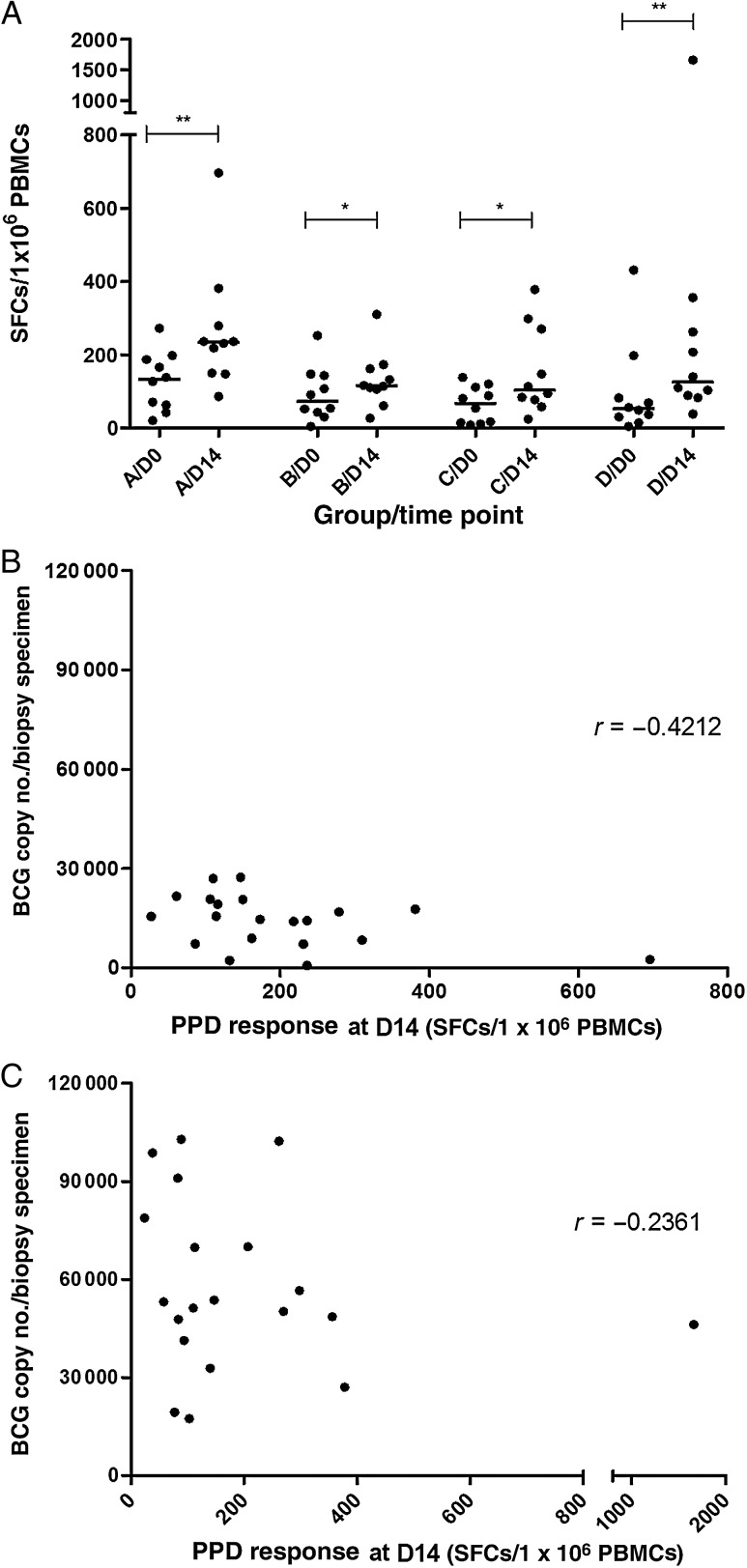
Ex vivo interferon γ enzyme-linked immunospot responses to stimulation with purified protein derivative (PPD; *A*) and relationship of day 14 PPD responses to the number of bacille Calmette-Guérin (BCG) detected in skin biopsy specimens by quantitative polymerase chain reaction from volunteers given a standard-dose challenge (*B*) and those given a high-dose challenge (*C*). Dots represent individual volunteers, and black lines show median values. *P < .05 and **P < .01, by the Mann–Whitney *U* test. Abbreviations: PBMC, peripheral blood mononuclear cell; SFC, spot-forming cell.

Responses to PPD were measured by ex vivo IFN-γ ELISpot analysis of fresh PBMCs from all volunteers at day 0 (the day of BCG challenge) and day 14 (the day of skin biopsy). Day 0 and day 14 responses did not differ significantly between the groups (by the Kruskal–Wallis test), suggesting that PPD response is not affected by strain or dose received. However, within each group, a significant increase in responses was observed between the 2 time points (Figure 4*A*) (*P* < .05, by the Wilcoxon matched-pairs signed rank test). PPD responses at day 14 showed a nonsignificant inverse correlation to the number of BCG detected by PCR in both the standard-dose (Figure 4*B*) and high-dose groups (Figure 4*C*). The same trend was observed with the CFU counts (data not shown), suggesting a trend toward a higher antigen-specific T-cell response resulting in a reduction in BCG copy number at the challenge site.

## DISCUSSION

We have evaluated a BCG challenge model to identify whether the strain or dose of the challenge agent can affect the sensitivity of the model. This has enabled us to select the most suitable combination of BCG dose and strain for the future use of the model.

In the challenge site skin biopsy specimens, significantly higher numbers of BCG were detected in the higher-dose groups, compared with the standard-dose groups, and the spread of detectable BCG was much greater. With regard to strain, there was no significant difference between the number of BCG detected between the high-dose SSI group and the high-dose TICE group.

Clinically, the adverse event profiles of all 4 challenge groups were acceptable, and there were no safety concerns from administering a higher dose of BCG in comparison to the standard dose of BCG administered in routine vaccination.

For future studies with this model, use of a high challenge dose would provide increased sensitivity to detect a difference between groups of volunteers who have different levels of vaccine-induced mycobacterial immunity, and so we conclude that a high dose of either strain would be acceptable for use in further studies.

Practical reasons favor the future use of high-dose SSI over high-dose TICE. BCG SSI is licensed for intradermal administration in the United Kingdom, and preparation of the vaccine for challenge is more straightforward, with less wastage of the product in comparison to BCG TICE, which requires a significant amount of dilution. Furthermore, we found that high-dose BCG SSI had less variability in dose across vials than high-dose BCG TICE across vials (the volunteers in group D tended to receive the greatest range of BCG dose, with a 10-fold difference between the lowest and highest dose given in the group). For future head-to-head testing of candidate vaccines with this model, we plan to challenge the same number of volunteers from each vaccine group with BCG from the same vial, to ensure as much standardization of challenge dose as possible.

Ex vivo IFN-γ ELISpot responses to PPD 2 weeks after challenge showed a trend toward an inverse correlation between the number of BCG detected in the skin biopsy specimens by qPCR and the number of CFU, consistent with our previous findings with this model [10]. This suggests that higher antigen specific T-cell responses lead to lower levels of BCG at the challenge site and demonstrates the utility of this model to facilitate the identification of potential immune correlates, which can be evaluated in field efficacy trials.

This skin BCG challenge model is also being tested and optimized for use in nonhuman primates (Harris et al unpublished data) and cattle [16]. An established BCG challenge model in these species would overcome the need for limited biosafety level 3 containment facilities required for virulent *M. tuberculosis* challenge. Furthermore, parallel skin BCG and lung *M. tuberculosis* challenge experiments in preclinical animal models can provide some validation of detection of a vaccine effect in the skin BCG challenge approach in human clinical trials.

Looking forward, we plan to use this optimized high dose BCG SSI challenge model to evaluate novel tuberculosis vaccine candidates. This model has utility to streamline the selection of which vaccines progress onto efficacy trials.

## Supplementary Data

Supplementary materials are available at http://jid.oxfordjournals.org. Consisting of data provided by the author to benefit the reader, the posted materials are not copyedited and are the sole responsibility of the author, so questions or comments should be addressed to the author.

Supplementary Data

## References

[1] World Health Organization (WHO). Global tuberculosis report 2014. Geneva: World Health Organization, 2014.

[2] Fletcher HA, Tanner R, Wallis RS et al Inhibition of mycobacterial growth in vitro following primary but not secondary vaccination with *Mycobacterium bovis* BCG. Clin Vaccine Immunol 2013; 20:1683–9. 23986316 10.1128/CVI.00427-13 PMC3837779

[3] Sauerwein RW, Roestenberg M, Moorthy VS Experimental human challenge infections can accelerate clinical malaria vaccine development. Nat Rev Immunol 2011; 11:57–64. 21179119 10.1038/nri2902

[4] Marwick C Volunteers in typhoid infection study will aid future vaccine development. JAMA 1998; 279:1423–4. 9600461 10.1001/jama.279.18.1423

[5] Luelmo F BCG vaccination. Am Rev Respir Dis 1982; 125:70–2. 7041722 10.1164/arrd.1982.125.3P2.70

[6] Lotte A, Wasz-Höckert O, Poisson N, Dumitrescu N, Verron M, Couvet E BCG complications. Estimates of the risks among vaccinated subjects and statistical analysis of their main characteristics. Adv Tuberc Res 1983; 21:107–93. 6475644

[7] Skeiky YA, Sadoff JC Advances in tuberculosis vaccine strategies. Nat Rev Microbiol 2006; 4:469–76. 16710326 10.1038/nrmicro1419

[8] Minassian AM, Ronan EO, Poyntz H, Hill AV, McShane H Preclinical development of an in vivo BCG challenge model for testing candidate TB vaccine efficacy. PLoS One 2011; 6:e19840. 21629699 10.1371/journal.pone.0019840 PMC3101220

[9] Minassian AM, Satti I, Poulton ID, Meyer J, Hill AV, McShane H A human challenge model for *Mycobacterium tuberculosis* using *Mycobacterium bovis* bacille Calmette-Guerin. J Infect Dis 2012; 205:1035–42. 22396610 10.1093/infdis/jis012 PMC3295601

[10] Harris SA, Meyer J, Satti I et al Evaluation of a human BCG challenge model to assess anti-mycobacterial immunity induced by BCG and a candidate TB vaccine, MVA85A, alone and in combination. J Infect Dis 2013; 209:1259–68. 24273174 10.1093/infdis/jit647 PMC3969545

[11] Hart PA, Sutherland I BCG and vole bacillus vaccines in the prevention of tuberculosis in adolescence and early adult life. BMJ 1977; 2:293–5. 326347 10.1136/bmj.2.6082.293 PMC1630784

[12] McShane H, Pathan AA, Sander CR et al Recombinant modified vaccinia virus Ankara expressing antigen 85A boosts BCG-primed and naturally acquired antimycobacterial immunity in humans. Nat Med 2004; 10:1240–4. 15502839 10.1038/nm1128

[13] Tameris MD, Hatherill M, Landry BS et al Safety and efficacy of MVA85A, a new tuberculosis vaccine, in infants previously vaccinated with BCG: a randomised, placebo-controlled phase 2b trial. Lancet 2013; 381:1021–8. 23391465 10.1016/S0140-6736(13)60177-4 PMC5424647

[14] Talbot EA, Williams DL, Frothingham R PCR identification of *Mycobacterium bovis* BCG. J Clin Microbiol 1997; 35:566–9. 9041390 10.1128/jcm.35.3.566-569.1997 PMC229628

[15] Meyer J, Harris SA, Satti I et al Comparing the safety and immunogenicity of a candidate TB vaccine MVA85A administered by intramuscular and intradermal delivery. Vaccine 2013; 31:1026–33. 23266342 10.1016/j.vaccine.2012.12.042 PMC5405058

[16] Dean G, Whelan A, Clifford D et al Comparison of the immunogenicity and protection against bovine tuberculosis following immunization by BCG-priming and boosting with adenovirus or protein based vaccines. Vaccine 2014; 32:1304–10. 24269321 10.1016/j.vaccine.2013.11.045

