# Supplementary material for: Optimization of a Human Bacille Calmette-Guérin Challenge Model: A Tool to Evaluate Antimycobacterial Immunity
Source: J Infect Dis. 2015 Oct 8;213(5):824–30. doi: 10.1093/infdis/jiv482 (PMC4747614; doi:10.1093/infdis/jiv482)
Supplement: Supplementary Data [file supp_213_5_824__index.html]

Optimisation of a human BCG challenge model: a tool to evaluate anti-mycobacterial immunity — Optimization of a Human Bacille Calmette-Guérin Challenge Model: A Tool to Evaluate Antimycobacterial Immunity — Optimization of a Human Bacille Calmette-Guérin Challenge Model: A Tool to Evaluate Antimycobacterial Immunity — Supplementary Data 

# Optimization of a Human Bacille Calmette-Guérin Challenge Model: A Tool to Evaluate Antimycobacterial Immunity

## Supplementary Data

Supplementary Data

- Supplementary Figure 1 - docx file
- Supplementary File 1 - docx file
- Supplementary File 2 - docx file
