# Supplementary material for: Optimization of a Human Bacille Calmette-Guérin Challenge Model: A Tool to Evaluate Antimycobacterial Immunity
Source: J Infect Dis. 2015 Oct 8;213(5):824–30. doi: 10.1093/infdis/jiv482 (PMC4747614; doi:10.1093/infdis/jiv482)
Supplement: Supplementary Data [file supp_jiv482_jiv482supp_fig1.docx]

**
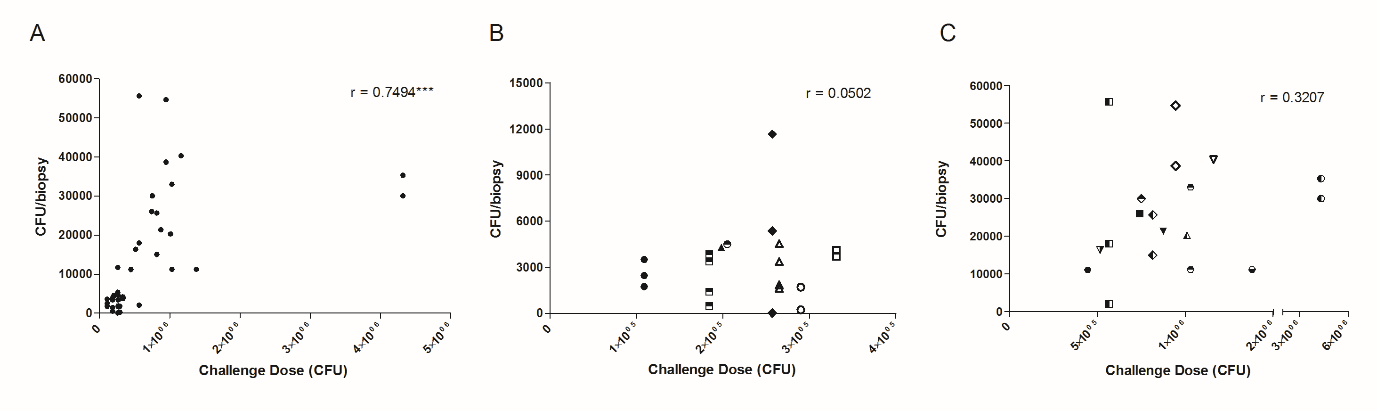
**

**Supplementary Figure 1. Correlation of BCG challenge dose received and recovery of BCG CFU from skin biopsies 14 days post-challenge in all 40 healthy BCG-naïve adults (A) and correlations when split into groups receiving standard (B) and high (C) dose BCG challenge.** Each symbol represents an individual volunteer, those volunteers with the same symbol in graphs B and C were challenged from the same vaccine vial (*** p < 0.001 Spearman Rho).
