# Supplementary material for: Optimization of a Human Bacille Calmette-Guérin Challenge Model: A Tool to Evaluate Antimycobacterial Immunity
Source: J Infect Dis. 2015 Oct 8;213(5):824–30. doi: 10.1093/infdis/jiv482 (PMC4747614; doi:10.1093/infdis/jiv482)
Supplement: Supplementary Data [file supp_jiv482_jiv482supp_file1.docx]

**Supplementary Methods 1. Full inclusion and exclusion criteria**

## Inclusion Criteria

Volunteers must meet all of the following criteria to enter the study:

- Healthy adult aged 18-55 years
- BCG naïve
- Resident in or near Oxford (for CCTVM) or Birmingham (for WTCRF) for the duration of the study period
- No relevant findings in medical history or on physical examination
- Allow the Investigators to discuss the volunteer’s medical history with their GP
- Use effective contraception for the duration of the study period (females only)
- Agreement to refrain from blood donation during the duration of the study
- Give written informed consent
- Allow the Investigator to register volunteer details with a confidential database to prevent concurrent entry into clinical trials
- Able and willing (in the Investigator’s opinion) to comply with all the study requirements

## Exclusion criteria

Volunteers must meet none of the following criteria to enter the study:

- Laboratory evidence at screening of latent M. *tb* infection as indicated by a positive ELISPOT response to ESAT6 or CFP10 antigens ^a^
- Clinical, radiological, or laboratory evidence of current active TB disease ^b^
- Previous vaccination with BCG, or any candidate TB vaccine
- Within the last year had close household contact with an individual with smear positive pulmonary tuberculosis
- Clinically significant history of skin disorder, allergy, immunodeficiency (including HIV), cancer (except BCC or CIS), cardiovascular disease, respiratory disease, gastrointestinal disease, liver disease, renal disease, endocrine disorder, neurological illness, psychiatric disorder, drug or alcohol abuse
- History of serious psychiatric condition
- Concurrent oral or systemic steroid medication or the concurrent use of other immunosuppressive agents
- History of anaphylaxis to vaccination or any allergy likely to be exacerbated by any component of the challenge agent
- Any abnormality of screening blood or urine tests that is deemed to be clinically significant or that may compromise the safety of the volunteer in the study^b^
- Positive HBsAg, HCV or HIV antibodies
- Female confirmed pregnant or intention to become pregnant during study period, or currently lactating
- Current involvement in another trial that involves regular blood tests or an investigational medicinal product^c^
- Use of an investigational medicinal product or non-registered drug, live vaccine, or investigational medical device for four weeks prior to dosing with the study challenge agent
- Administration of immunoglobulins and/or any blood products within the three months preceding the planned challenge date
- Any other significant disease, disorder, or finding, which, in the opinion of the Investigator, may either put the volunteer at risk, or may influence the result of the study, or may affect the volunteer’s ability to participate in the study

^a^ Volunteers discovered to have evidence of latent M. *tb* infection as defined by a positive ELISPOT test will be referred for a plain chest x-ray. If there is any evidence of active TB disease either on clinical or radiological grounds, further investigation and treatment will be offered under the supervision of a consultant physician in respiratory medicine or infectious diseases.

^b^ Volunteers who are excluded from the study because they have been discovered to have a previously undiagnosed condition thought to require further medical attention will be referred appropriately to their GP or an NHS specialist service for further investigation and treatment.

^c^ Volunteers will be excluded from the study if they are concurrently involved in another trial that involves regular blood tests or an investigational medicinal product. In order to check this, volunteers will be asked to provide their National Insurance or Passport number (if they are not entitled to a NI number) and will be registered on a national database of participants in clinical trials ([www.tops.org.uk](http://www.tops.org.uk)).
