# Supplementary material for: Optimization of a Human Bacille Calmette-Guérin Challenge Model: A Tool to Evaluate Antimycobacterial Immunity
Source: J Infect Dis. 2015 Oct 8;213(5):824–30. doi: 10.1093/infdis/jiv482 (PMC4747614; doi:10.1093/infdis/jiv482)
Supplement: Supplementary Data [file supp_jiv482_jiv482supp_file2.docx]

**Supplementary Methods 2. Dilution to achieve desired dose of BCG**

BCG: Bacillus Calmette–Guérin

**Group A. BCG SSI 2 - 8 x 10^5^ pfu**

- *Supplied as: 1 vial SSI reconstituted in 1mL contains 10 doses of 2 - 8 x 10^5^ pfu (*taken as 5x10^5^ pfu for this calculation)*
- For the specified volume of 0.15mL to contains the specified dose of 2 - 8 x 10^5^ pfu the solution needs to be diluted to a concentration of 2 - 8 x 10^5^ pfu/1.5mL (0.15mL x 10 =1.5mL)

i.e 1.5mL - 1mL = 0.5mL

- Therefore, if 0.5mL of saline is added to the reconstituted BCG SSI:

150 microlitres contains 2 - 8 x 10^5^ pfu

**Group B. BCG TICE 2 - 8 x 10*^5^* pfu**

- *Supplied as: 1 vial TICE in 1mL saline contains 2 - 8x10^8^ pfu (*taken as 5.0x10^8^ for this calculation)*
- To attain the specified dose of 2 - 8 x 10*^5^* pfu (*taken as 5 x 10*^5^* pfu for this calculation) in the specified volume of 0.15mL, the solution needs to be diluted to the following concentration:

[required concentration = desired dose/ desired volume]

[3.3x10^6^ pfu/mL = 5 x 10*^5^* pfu/0.15mL]

- To attain the required concentration from the supplied concentration the dilution factor needs to be calculated:

[DF = current concentration/desired concentration]

[5.0x10^8^ pfu/mL ÷ 3.3x10^6^ pfu/mL = dilution factor of 150]

- To achieve a final dilution factor of 1/150, serial dilutions have to be carried out according to the following formula:

[D_(final)_ = D_A_ x D_B_ x etc]

Staring with 1/10 dilutions (ie. If D_A_ = 1/10 and D_B_ = 1/10):

D_C_ = D_(final)_ / (D_A_ x D_B_)

D_C_ = 1/150 / (1/10 x 1/10)

D_C_ = 6.7/10

Therefore, if we dilute (1/10 x 1/10 x 6.7/10) i.e. 1mL in 9mL, then 1mL of this in 9mL, then 6.7ml of this in 3.3mL we have a 1/150 dilution.

150 microlitres contains 2 - 8 x 10^5^ pfu

**Group C. BCG SSI 6 - 24 x 10^5^ pfu**

- *Supplied as: 1 vial SSI reconstituted in 1mL contains 10 doses of 2 - 8 x 10^5^ pfu (*taken as 5x105 pfu for this calculation)*

For the specified volume of 0.15mL to contain the specified dose of 6 - 24 x 10^5^ pfu the solution needs to be diluted to a concentration of 6 - 24 x 10^5^ pfu/0.5mL

Therefore, if the SSI is reconstituted in 0.5mL diluent (1mL x 0.5 = 0.5mL)

150 microlitres contains 6 - 24 x 10^5^ pfu

**Group D. BCG TICE 6 - 24 x 10^5^ pfu**

*Supplied as: 1 vial TICE in 1mL saline contains 2 - 8x10^8^ pfu (*taken as 5.0x10^8^ for this calculation)*

- To attain the specified dose of 6 - 24 x 105 pfu (*taken as 15 x 10^5^ pfu for this calculation) in the specified volume of 0.15mL, the solution needs to be diluted to the following concentration:

[required concentration = desired dose/ desired volume]

[1x10^7^pfu/mL = 15 x 10^5^ pfu/0.15mL]

- To attain the required concentration from the supplied concentration the dilution factor needs to be calculated:

[D_F_ = current concentration/desired concentration]

[5.0x10^8^ pfu/mL ÷ 1x10^7^ pfu/mL = dilution factor of 50]

- To achieve a final dilution factor of 1/50, serial dilutions have to be carried out according to the following formula:

[D_(final)_ = D_A_ x D_B_ x etc]

Staring with a 1/10 dilutions (ie. If D_A_ = 1/10):

D_B_ = D_(final)_ / D_A_

D_B_ = 1/50 ÷ 1/10

D_B_ = 2.0/10

Therefore, if we dilute (1/10 x 2.0/10) i.e. 1mL in 9mL, then 2.0mL of this in 8.0mL, we have a 1/50 dilution

150 microlitres contains 6 - 24 x 10^5^ pfu
